# The C242T polymorphism of the p22-phox gene (CYBA) is associated with higher left ventricular mass in Brazilian hypertensive patients

**DOI:** 10.1186/1471-2350-12-114

**Published:** 2011-08-31

**Authors:** Roberto Schreiber, Maria C Ferreira-Sae, Juliana A Ronchi, José A Pio-Magalhães, José A Cipolli, José R Matos-Souza, José G Mill, Aníbal E Vercesi, José E Krieger, Kleber G Franchini, Alexandre C Pereira, Wilson Nadruz Junior

**Affiliations:** 1 Department of Internal Medicine, University of Campinas, Campinas, Brazil; 2 Department of Clinical Pathology, University of Campinas, Campinas, Brazil; 3 Federal University of Espírito Santo, Vitória, Brazil; 4 Laboratory of Genetics and Molecular Cardiology, Heart Institute (InCor), University of São Paulo, São Paulo, Brazil

**Keywords:** p22-phox, left ventricle, hypertension, polymorphism, NADPH-oxidase

## Abstract

**Background:**

Reactive oxygen species have been implicated in the physiopathogenesis of hypertensive end-organ damage. This study investigated the impact of the C242T polymorphism of the p22-phox gene (CYBA) on left ventricular structure in Brazilian hypertensive subjects.

**Methods:**

We cross-sectionally evaluated 561 patients from 2 independent centers [Campinas (n = 441) and Vitória (n = 120)] by clinical history, physical examination, anthropometry, analysis of metabolic and echocardiography parameters as well as p22-phox C242T polymorphism genotyping. In addition, NADPH-oxidase activity was quantified in peripheral mononuclear cells from a subgroup of Campinas sample.

**Results:**

Genotype frequencies in both samples were consistent with the Hardy- Weinberg equilibrium. Subjects with the T allele presented higher left ventricular mass/height^2.7 ^than those carrying the CC genotype in Campinas (76.8 ± 1.6 vs 70.9 ± 1.4 g/m^2.7^; p = 0.009), and in Vitória (45.6 ± 1.9 vs 39.9 ± 1.4 g/m^2.7^; p = 0.023) samples. These results were confirmed by stepwise regression analyses adjusted for age, gender, blood pressure, metabolic variables and use of anti-hypertensive medications. In addition, increased NADPH-oxidase activity was detected in peripheral mononuclear cells from T allele carriers compared with CC genotype carriers (p = 0.03).

**Conclusions:**

The T allele of the p22-phox C242T polymorphism is associated with higher left ventricular mass/height^2.7 ^and increased NADPH-oxidase activity in Brazilian hypertensive patients. These data suggest that genetic variation within NADPH-oxidase components may modulate left ventricular remodeling in subjects with systemic hypertension.

## Background

Left ventricular (LV) hypertrophy is an independent predictor of cardiovascular events and is a major risk factor for the development of heart failure in hypertensive subjects [1]. Oxidative stress derived from Nicotinamide adenine dinucleotide phosphate (NADPH)-oxidase has been implicated in the physiopathogenesis of hypertensive LV remodeling [2,3]. Among the subunits that comprise the NADPH-oxidase system, the p22-phox is highlighted as an essential membrane-associated factor that plays a crucial role in the activation and stabilization of this enzymatic complex [4]. In this regard, experimental evidence showed that LV hypertrophy is accompanied by increased myocardial p22-phox expression in aortic-banded rats, suggesting that this protein might be involved in hypertensive cardiac remodeling [2,5].

To date, several polymorphisms of the p22-phox gene (CYBA) have been identified [6]. One of the most studied polymorphisms of this gene is the C242T, which predicts the nonconservative substitution of histidine-72 by a tyrosine residue [7] and has been shown to enhance the functional activity of NADPH-oxidase [8]. However, although numerous studies investigated the role of the p22-phox C242T polymorphism in cardiovascular phenotypes [9], a significant heterogeneity for a modulating role of the T allele has been reported [9,10]. In addition, little is known about the impact of this variant on the development of hypertensive end-organ damage. Thus, the aim of the present report was to investigate whether the C242T p22-phox polymorphism is associated with variation in LV structure in hypertensive subjects.

## Methods

### Study population

The study was carried out in 561 unrelated hypertensive subjects from two independent centers located in distinct states of Brazil. The Campinas sample consisted of hypertensive patients from a tertiary referral clinic hospital and comprised 441 subjects (264 women and 177 men) from the city of Campinas, São Paulo State, with high prevalence of end-organ damage [11,12]. The Vitória sample consisted of hypertensive patients from a population-based study and comprised 120 subjects (76 women and 44 men) from the city of Vitória, Espírito Santo State [13]. The research was carried out in accordance with the Declaration of Helsinki of the World Medical Association. This study was approved by the Human Research Ethics Committee of the University of Campinas and by the Committee for Research on Human Subjects of the Espírito Santo Federal University. All subjects gave written informed consent to participate.

Hypertension was defined as systolic blood pressure ≥ 140 mmHg or diastolic blood pressure ≥ 90 mmHg or current antihypertensive medication use. Diabetes mellitus was diagnosed if fasting blood glucose was ≥ 126 mg/dL or when participants were taking hypoglycemic medications [14]. Coronary heart disease was diagnosed by history of myocardial infarction, acute coronary syndrome or coronary revascularization or by evidence of cardiac ischemia documented by functional testing. Main exclusion criteria were age under 18 years, significant cardiac valve disease, hypertrophic cardiomyopathy and neoplastic disease.

Blood pressure was measured using a validated digital oscillometric device (HEM-705CP; Omron Healthcare, Kyoto, Japan) with appropriate cuff sizes. Two readings were averaged and, if they differed by more than 5 mmHg, one additional measurement was performed and the average of the three measurements was taken.

Body mass index was calculated as body weight divided by height squared (kg/m^2^). Fasting blood total cholesterol, low-density-lipoprotein cholesterol, high-density-lipoprotein cholesterol, triglycerides, uric acid and glucose levels were measured using standard laboratory techniques. In addition, creatinine clearance was calculated from serum and urine samples.

### Echocardiography

Echocardiography studies were performed on each subject at rest in the left lateral decubitus position using a Vivid 3 Pro (General Electric, Milwaukee, WI) apparatus equipped with a 2.5 MHz transducer as previously described [15,16]. LV end-diastolic and end-systolic diameters, interventricular septum thickness, posterior wall thickness and LV mass were measured in accordance with the American Society of Echocardiography guidelines [17]. Relative wall thickness was computed as twice the posterior wall thickness divided by LV end-diastolic diameter. LV mass index was considered as LV mass/height^2.7^. All the recordings were made by one physician in the Campinas sample and by another physician in the Vitória sample. The reproducibility of both acquiring and measuring LV mass was determined in recordings obtained from 10 subjects in each sample. Intraobserver and interobserver LV mass variabilities were < 8% and < 11%, respectively.

### Genotyping

Genomic DNA was extracted from peripheral blood leukocytes. The p22-phox C242T polymorphism was analyzed by polymerase chain reaction and digestion with Rsa-I restriction enzyme [18]. Quality control for these assays was assessed by randomly selecting 40 samples to be re-genotyped by two independent technicians.

### NADPH-oxidase activity assay

NADPH-oxidase activity was assessed in peripheral mononuclear cells (monocytes and lymphocytes) isolated from blood samples according to Degasperi et al. [19]. Briefly, peripheral blood monocytic cells from 18 hypertensive patients of the Campinas sample carrying different p22-phox C242T genotypes (6 CC, 6 CT and 6 TT subjects) were isolated by density gradient centrifugation over Histopaque 1077 and washed twice with phosphate-buffered saline. Cells were counted in a Neubauer chamber, and cell viability was determined by trypan blue exclusion method. Cells were only used when viability was greater than 98%. In order to evaluate NADPH-oxidase activity, dihydroethidium (DHE) (Molecular Probes Inc., Eugene, OR) was used as a sensitive probe. For this purpose, cells (2 × 10^6 ^cells/mL) were immediately incubated with 10 μM DHE at 37°C, with or without 10 μM diphenylene iodonium (DPI), a selective inhibitor of NADPH-oxidase, and the fluorescence intensity was quantified in a spectrofluorometer (F-4500, Hitachi, Tokyo, Japan) using excitation and emission wavelengths of 563 and 587 nm, respectively, and slit widths of 5 nm, with gentle continuous stirring. The difference between the activities measured without and with DPI was considered as NADPH-oxidase activity.

### Statistical analysis

Data were analyzed using SPSS 15.0™. Descriptive statistical results are given as means ± SEM. The differences in genotype distributions, categorical variables as well as Hardy-Weinberg disequilibria for polymorphism were tested using chi-square test. Unpaired t-test was used to compare continuous variables and to evaluate differences in NADPH-oxidase activity between genotype groups. Stepwise regression analyses evaluated the independent predictors of LV mass index. A *p-*value of less than 0.05 was considered significant.

## Results

### Distribution and allele frequency of polymorphism

The distribution of genotypes and the frequency of alleles of the polymorphism C242T of the p22-phox gene are summarized in Table 1. The allele frequencies in both samples obeyed the Hardy-Weinberg's law. There was no significant difference in the genotype distribution between Campinas and Vitória samples (X^2 ^= 1.6815; p = 0.4314).

**Table 1:** p22-phox C242T polymorphism in patients with hypertension

Genotype	Campinas, n (%)	Vitória, n (%)	
TT	60 (13.6)	11 (9.2)	
CT	189 (42.8)	54 (45.0)	
CC	192 (43.6)	55 (45.8)	
T Allele frequency	0.35	0.32	p = 0.431
C Allele frequency	0.65	0.68	X^2 ^= 1.681

### Sample characteristics

The clinical and laboratory characteristics of hypertensive subjects in both samples according to the C242T polymorphism are shown in Table 2. Given the described dominant effect of the T allele in functional and association studies [8,20-22] and in order to enhance statistical power, TT subjects were added to CT ones. No differences regarding the clinical and laboratory features were detected between the genotype groups in both samples, except for higher diabetes mellitus prevalence and glucose levels in Campinas CT+TT subgroup and higher height in Vitória CC group.

**Table 2:** Characteristics of hypertensive subjects according to the C242T polymorphism

Variable	Campinas		p	Vitória		p
Genotype (N)	CT + TT (249)	CC (192)		CT + TT (65)	CC (55)	
Age, years	57.2 ± 0.8	56.4 ± 0.9	0.573	54.2 ± 1.2	57.3 ± 1.2	0.078
Gender (M/F)	101/148	76/116	0.912	20/45	24/31	0.205
Weight, kg	80 ± 1	77 ± 1	0.075	80 ± 1	80 ± 2	0.727
Height, cm	160 ± 1	159 ± 1	0.318	158 ± 1	161 ± 1	0.015
Body mass index, kg/m^2^	31.5 ± 0.3	30.6 ± 0.4	0.118	31.9 ± 0.4	30.9 ± 0.5	0.143
Systolic BP, mmHg	148 ± 1	146 ± 1	0.457	143 ± 2	144 ± 2	0.702
Diastolic BP, mmHg	85 ± 1	86 ± 1	0.762	91 ± 2	94 ± 2	0.359
Diabetes mellitus, n (%)	81 (32)	40 (21)	0.008	10 (15)	6 (11)	0.653
Smokers, n (%)	25 (10)	21 (11)	0.885	14 (21)	7 (13)	0.305
Coronary heart disease, n (%)	36 (14)	34 (18)	0.426	2 (3)	2 (4)	0.864
LDL-cholesterol, mg/dL	112 ± 2	112 ± 2	0.829	124 ± 5	117 ± 4	0.291
HDL-cholesterol, mg/dL	51 ± 1	52 ± 1	0.748	43 ± 1	45 ± 1	0.401
Triglycerides, mg/dL	162 ± 6	148 ± 6	0.100	195 ± 13	182 ± 13	0.501
Glucose, mg/dL	116 ± 3	106 ± 3	0.030	117 ± 6	106 ± 6	0.184
Uric acid, mg/dL	5.9 ± 0.1	6.1 ± 0.1	0.344	5.5 ± 0.1	5.4 ± 0.1	0.779
Creatinine clearance rate, mL/min	88 ± 2	91 ± 2	0.409	88 ± 4	86 ± 4	0.733
Diuretics, n (%)	188 (75)	154 (80)	0.289	26 (40)	25 (45)	0.676
Calcium channel blockers, n (%)	124 (50)	97 (51)	0.956	8 (12)	7 (13)	0.944
Beta-Blockers, n (%)	117 (47)	86 (45)	0.717	15 (23)	13 (24)	0.942
ACEI or ARB, n (%)	191 (77)	153 (79)	0.526	32 (49)	19 (34)	0.151

BP - blood pressure; LDL - low-density lipoprotein; HDL - high-density lipoprotein; ACEI or ARB - angiotensin-converting enzyme inhibitors or angiotensin receptor blockers.

### Association of the C242T polymorphism with LV structure

Echocardiography data are demonstrated in Table 3. Hypertensive subjects carrying the T allele of the C242T polymorphism presented higher LV mass/height^2.7 ^than those with the CC genotype in both samples. In addition subjects from the Campinas sample with the T allele exhibited higher posterior wall thickness and LV end-diastolic diameter, compared to CC genotype carriers.

**Table 3:** Echocardiographic features of hypertensive patients according to the C242T polymorphism

Variable	Campinas		p	Vitória		p
Genotype (N)	CT + TT (249)	CC (192)		CT + TT (65)	CC (55)	
Interventricular septum, mm	10.9 ± 0.1	10.7 ± 0.1	0.089	9.1 ± 0.1	8.8 ± 0.1	0.141
Posterior wall thickness, mm	10.9 ± 0.1	10.5 ± 0.1	0.010	8.8 ± 0.1	8.7 ± 0.1	0.402
LV end-diastolic diameter, mm	50.9 ± 0.4	49.7 ± 0.4	0.047	48.8 ± 0.7	47.8 ± 0.6	0.272
Relative wall thickness	0.433 ± 0.004	0.425 ± 0.005	0.387	0.364 ± 0.005	0.362 ± 0.006	0.825
LV Mass/height ^2.7^, g/m^2.7^	76.8 ± 1.6	70.9 ± 1.4	0.009	45.6 ± 1.9	39.9 ± 1.4	0.023

LV - left ventricular.

Considering that the relationships between the C242T polymorphism and LV mass/height^2.7 ^could be influenced by potential confounders, stepwise regression analyses were performed (Table 4). The 242T allele was independently related to LV mass/height^2.7 ^in both samples by using a model that included gender, age, body mass index, systolic blood pressure, diastolic blood pressure, p22-phox C242T polymorphism, diabetes mellitus, calcium channel blockers use and angiotensin-converting enzyme inhibitors/angiotensin receptor blockers use as independent variables.

**Table 4:** Stepwise regression analyses for LV mass index (g/m^2.7^)

Step	Variable	R^2 ^Change	F Ratio	P
	Model 1: Campinas sample			
1	Body mass index	0.086	41.0	< 0.00001
2	Age	0.040	19.7	0.00001
3	p22-phox C242T (CC = 0; CT+TT = 1)	0.011	4.8	0.029
4	Systolic blood pressure	0.010	4.5	0.036
				
	Model 2: Vitória sample			
1	Gender	0.067	8.5	< 0.004
2	Diastolic blood pressure	0.036	4.7	0.032
3	p22-phox C242T (CC = 0; CT+TT = 1)	0.032	4.3	0.041

Both models included gender, age, body mass index, systolic blood pressure, diastolic blood pressure, p22-phox C242T polymorphism, diabetes mellitus, calcium channel blockers use and angiotensin-converting enzyme inhibitors/angiotensin receptor blockers use as independent variables. Only variables with significant association were presented. LV - left ventricular.

### Relationship between NADPH-oxidase activity and the C242T polymorphism

**Figure 1:**
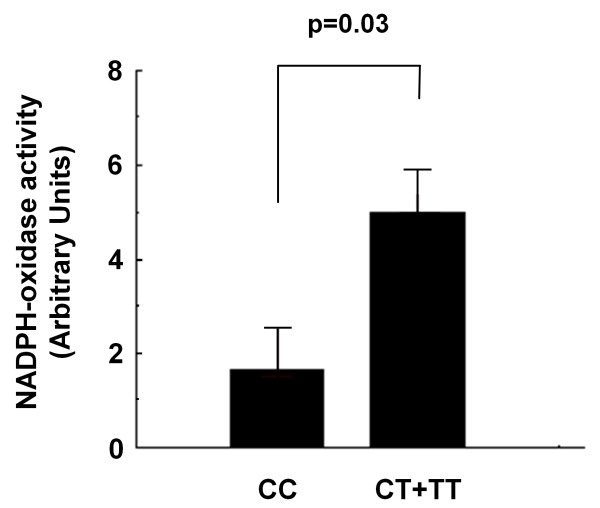
**NADPH-oxidase activity in peripheral blood mononuclear cells from hypertensive subjects according to the p22-phox C242T polymorphism**.

To investigate the effects of the polymorphism on the NADPH-oxidase activity, we used DHE as a sensitive probe to evaluate superoxide anion production in peripheral blood mononuclear cells from 18 hypertensive patients of the Campinas sample (Figure 1). Mononuclear cells from subjects with the T allele (n = 12) presented higher NADPH-oxidase activity (p = 0.03) than those from individuals carrying the CC genotype (n = 6).

## Discussion

Experimental evidence has indicated that activation of the NADPH-oxidase plays a major role in the development of LV hypertrophy induced by pressure overload [2,3]. In the present report we found that the p22-phox C242T variant was independently associated with higher LV mass index in hypertensive subjects from 2 distinct Brazilian populations. Furthermore, *in vitro *assays demonstrated that peripheral blood mononuclear cells of T allele carriers presented higher NADPH-oxidase activity, compared to non-carriers. Taken together, these findings suggest that a functional p22-phox polymorphism is associated with alterations in LV mass in hypertensive subjects, thus extending to humans the notion that variation in NADPH-oxidase activation might influence LV remodeling induced by pressure overload.

Several studies investigated the role of the p22-phox C242T polymorphism in cardiovascular phenotypes. However, a significant heterogeneity for a modulating role of the T allele in the occurrence of atherothrombotic disease and hypertension has been reported [9,10]. In this context, carriers of the 242T allele were reported to be more susceptible to coronary artery disease [20], cerebrovascular disease [21] and hypertension [22], while other groups described neutral or even protective effects of this variant against such conditions [23-26]. Conversely, the role of the C242T polymorphism as well as of other p22-phox variants in the development of hypertensive end-organ damage is poorly understood. Previous data from our group demonstrated that the -930AG polymorphism of the p22-phox was not associated with alterations in LV structure and renal damage in Brazilian hypertensive subjects [27]. In the present report, we provided novel evidence that the C242T polymorphism was associated with higher LV mass index in hypertensive patients independently of potential confounding factors such as age, gender, blood pressure, metabolic variables and use of anti-hypertensive medications. Noticeably these findings were reproduced in 2 independent and unrelated samples, with quite different clinical features, thus strengthening the validity of the results. In this regard, the Campinas sample comprised subjects with high prevalence of end-organ damage who attended a tertiary hospital, while the Vitória sample included individuals from a population-based study. On the other hand, given that our functional assays demonstrated higher NADPH-oxidase activity in mononuclear cells carrying the T allele, it can be speculated that functional genetic variation in NADPH-oxidase components may modulate LV remodeling in hypertensive patients. Nevertheless, it cannot be discarded that the C242T polymorphism is in linkage disequilibrium with another functional polymorphism, located in the same gene or in its vicinity.

The present study showed that NADPH-oxidase activity of mononuclear cells with the T allele was higher than that of mononuclear cells without the T allele, which indicates that the C242T polymorphism of p22-phox might lead to gain of function for the NADPH-oxidase activity. Other reports previously assessed the functional role of this polymorphism in leukocyte NADPH-oxidase activity, but yielded conflicting results. For instance, studies using phorbol 12-myristate 13-acetate (PMA) stimulation have demonstrated either increased [8] or reduced [26,28] NADPH-oxidase activity in leucocytes carrying the T allele. The reasons for such discrepancies are not apparent but it is possible that differences in clinical and ethnical features among the studied populations might have played a role in this regard. Conversely, although considered a stimulus for NADPH-oxidase activation [8,26,28], PMA also induces mitochondrial release of reactive oxygen species [29], which could also account for the discrepant results. Here, evaluation of NADPH-oxidase activation was performed by treating mononuclear cells with DPI, which is considered a specific inhibitor of NADPH-oxidase [30], thus differing from the aforementioned studies. In addition, our findings agree with data showing increased superoxide production in transgenic HL-60 cells transfected with expression plasmids carrying p22-phox cDNAs with C242T polymorphism [8], thus strengthening the notion that this variant might induce gain of function for the NADPH-oxidase activity.

One limitation of this study is that part of hypertensive subjects was on medications. Some findings regarding the impact of the polymorphism on LV mass might be, therefore, attributable to differential effect of various therapy regimens. However, we diminished this potential bias by considering in multivariate models the presence of antihypertensive medications. On the other hand, it should be acknowledged that some variables that may modulate LV remodeling, such as duration of hypertension and diabetes control, were not included in multivariate analysis. This might constitute another potential limitation to this study.

## Conclusions

This study shows that genetic variation within p22-phox gene is significantly associated with higher LV mass in two independent Brazilian samples of hypertensive patients. A more complete understanding of the biochemical processes by which p22-phox alters myocardial growth may aid in unveiling how genetic variation in NADPH-oxidase components might modulate LV remodeling in hypertensive patients.

## Competing interests

The authors declare that they have no competing interests.

## Authors' contributions

RS carried out the molecular genetic studies, statistical analysis and drafted the manuscript. MCFS carried out the molecular genetic studies and participated in individual selection and characterization. JAR and AEV carried out the NADPH-oxidase activity assay. JAC and JGM were responsible for echocardiography studies. JAPM, JRMS, JEK and KGF participated in the design of the study and were responsible for individual selection and characterization. WNJ and ACP participated in the design of the study, statistical analysis and coordinated experiments and manuscript preparation. All authors read and approved the final manuscript.

## Pre-publication history

The pre-publication history for this paper can be accessed here:

http://www.biomedcentral.com/1471-2350/12/114/prepub
